# Endophytic Seed-Associated Bacteria as Plant Growth Promoters of Cuban Rice (*Oryza sativa* L.)

**DOI:** 10.3390/microorganisms11092317

**Published:** 2023-09-14

**Authors:** Ionel Hernández, Cecilia Taulé, Reneé Pérez-Pérez, Federico Battistoni, Elena Fabiano, Angela Villanueva-Guerrero, María Caridad Nápoles, Héctor Herrera

**Affiliations:** 1 National Institute of Agricultural Science, Plant Physiology and Biochemistry Department, Carretera a Tapaste Km 3 y ½, San José de las Lajas 32700, Mayabeque, Cuba; renee.pp.2012@gmail.com (R.P.-P.); mariacaridad.napoles@gmail.com (M.C.N.); 2 Biological Research Institute Clemente Estable, Microbial Biochemistry and Genomics Department, Avenida Italia 3318, Montevideo 11600, Uruguay; cecilia.taule@gmail.com (C.T.); febattist@gmail.com (F.B.); elena.fabiano@gmail.com (E.F.); 3 Laboratorio de Silvicultura, Departamento de Ciencias Forestales, Facultad de Ciencias Agropecuarias y Medioambiente, Universidad de La Frontera, Temuco 4811230, Chile; angela.villanueva@ufrontera.cl; 4 Programa de Magister en Manejo de Recursos Naturales, Facultad de Ciencias Agropecuarias y Medioambiente, Universidad de La Frontera, Temuco 4811230, Chile; 5 Laboratorio de Ecosistemas y Bosques, Departamento de Ciencias Forestales, Facultad de Ciencias Agropecuarias y Medioambiente, Universidad de La Frontera, Temuco 4811230, Chile

**Keywords:** biocontrol, endophytes, *Pantoea*, plant growth promotion, *Pseudomonas*, symbiosis

## Abstract

Cuban rice cultivars INCA LP-5 and INCA LP-7 are widely distributed in Cuba and Caribbean countries. Although there are studies about rhizospheric bacteria associated with these cultivars, there are no reports about their seed-associated bacteria. This study aimed to isolate endophytic bacteria from rice seeds and select those with the greatest plant growth-promoting traits. A total of nineteen bacterial strains from the genera *Pantoea*, *Bacillus*, *Paenibacillus*, and *Pseudomonas* were isolated from the husk and endosperm of rice seeds. The strains *Pantoea* sp. S5-1, *Pseudomonas* sp. S5-38, and *Pseudomonas* sp. S7-1 were classified as the most promissory to increase rice growth as they demonstrated the presence of multiple plant growth-promoting traits such as the production of auxins, phosphate, and potassium solubilization, the production of siderophores, and the inhibition of the phytopathogen *Pyricularia oryzae*. The inoculation of strains of *Pantoea* sp. and *Pseudomonas* spp. in rice improves the height, root length, fresh weight, and dry weight of the shoot and root after 21 days post-inoculation in hydroponic assays. This study constitutes the first report on Cuban rice cultivars about the presence of endophytes in seeds and their potential to promote seedling growth. *Pantoea* sp. S5-1, *Pseudomonas* sp. S5-38, and *Pseudomonas* sp. S7-1 were selected as the more promising strains for the development of bio-stimulators or bio-inoculants for Cuban rice crops.

## 1. Introduction

Rice (*Oryza sativa* L.) is one of the most important crops worldwide. Its production reached around 519.5 million tons in the 2022–2023 season [1]. In Cuba, rice is a prioritized crop since annual consumption is 70 kg per capita, one of the highest in Latin America and representing the average per capita consumption of Asian countries [2,3]. However, the national rice production (226 thousand tons) does not satisfy all the country’s demands, and more than half of the consumed rice is imported from Vietnam, Brazil, and other Southeast Asian countries [2,3]. Soil depletion, fertilizers, and pesticide shortages, lack of labor force, high production costs, few planted areas, and progressive drought linked to the effects of climate change are some of the problems with a negative impact on rice production in Cuba [2,3]. Consequently, the search for alternatives to improve the local production of rice under challenging environmental conditions is vital for satisfying the internal rice demands.

Cuban rice cv. INCA LP-5 and INCA LP-7 are part of the eleven principal rice cultivars planted in Cuba [4]. They have high yield potential and moderate resistance to the phytopathogens *Pyricularia oryzae* and *Tagosodes orizicolus* [5]. Additionally, the cultivar cv. INCA LP-7 is tolerant to salinity and resistant to *Polyphagotarsonemus latus* (white mite) [6]. Both cultivars are distributed in 33% of the area destined for rice production (approximately 3.000 ha), which is a significant surface since the other nine cultivars are planted in the remaining 67% of the planted areas [4]. Therefore, there is a great interest in increasing rice yield, especially with a low input of chemical fertilizers.

The use of plant growth-promoting bacteria (PGPB) is an environmentally friendly strategy for crop production [7]. The beneficial effect on plant growth induced by PGPB is based on a set of direct and indirect mechanisms that act simultaneously [8]. The direct mechanism of growth promotion includes the production of metabolites that improve plant growth (e.g., phytohormones, siderophore, and enzymes), solubilization of essential nutrients (e.g., nitrogen, phosphorous, potassium, and iron), and regulating the gene expression on the plant to improve the responses to environmental stress [9,10,11], while indirect mechanisms are mainly based on biocontrol activity against phytopathogens [12].

Endophytic PGPB live asymptomatically inside vital plant organs and comprise bacteria acquired from seeds (vertically obtained from the mother plants) or soils (horizontally acquired from the environment) [13,14]. Seed endophytic bacteria play a vital role in the germination, resistance of the seedlings to changes in climate conditions, maintaining plant health, nutrient mobilization, control of phytopathogens, antioxidant activity, and hormone production [15,16,17]. Bacteria belonging to the phyla Actinobacteria, Bacteroidetes, and Firmicutes are commonly transmitted and survive in the plant seeds [18]. Interestingly, rice seeds host beneficial bacteria that spread into the roots and shoots and are transmitted to the next generations [19]. Previous studies have demonstrated that rice seeds harbor a specific bacterial endophytic microbiota, including bacterial genera such as *Sphingomonas*, *Flavobacterium*, *Microbacterium*, and *Pseudomonas* [20,21]. Despite the interaction of rice with beneficial microorganisms reported, there is unknown to us the diversity of bacterial endophytes associated with rice seeds and how they can support plant growth at early stages of development is mainly unknown.

In Cuba, the PGPB community associated with rice rhizosphere is mainly composed of species belonging to the genera *Azospirillum*, *Herbaspirillum*, *Bacillus*, *Burkholderia*, *Pseudomonas,* and *Azotobacter* [22,23,24]. Moreover, bacterial strains belonging to the genera *Rhizobium*, *Enterobacter*, *Pantoea*, *Mitsuaria*, *Acinetobacter*, and *Pseudomonas* were recently reported in the rhizosphere of Cuban rice cultivars INCA LP-5 and INCA LP-7 [25,26]. However, it is unknown how seed endophytic bacteria associated with Cuban rice can enhance plantlet growth. Therefore, we hypothesized that Cuban rice seeds harbor an endophytic bacterial community that can support growth at the plantlet stage. Therefore, this study aimed to isolate bacteria from rice seeds and select those with the greatest potential for promoting plant growth. As far as we know, there is no previous evidence about endophytic bacteria associated with seeds of Cuban rice cultivars.

## 2. Materials and Methods

### 2.1. Isolation and Identification of Endophytic Seed-Associated Bacteria 

Fresh certified rice cv. INCA LP-5 and INCA LP-7 seeds were acquired from the Basic Technological-Scientific Unit “Los Palacios” in Pinar del Rio, Cuba. One gram of seeds with husk was surface disinfected, according to García et al. [27], using 70% (*v*/*v*) ethanol for 1 min and a calcium hypochlorite solution (10 g calcium hypochlorite, 500 μL Tween 80, 200 mL distilled water) for 20 min, while one gram of seeds without husk was surface-disinfected with 70% (*v*/*v*) ethanol for 5 min, kept in 6% (*v*/*v*) sodium hypochlorite for 15 min, and washed 20 times with sterile distilled water. The efficiency of superficial disinfection was assessed by placing 100 μL of the final rinse onto a tryptone–yeast extract (TY) medium (5.0 g·L^−1^ tryptone, 3.0 g·L^−1^ yeast extract, 0.68 g·L^−1^ CaCl_2_) and a further 48 h of incubation at 30 °C. The absence of colonies was indicative of proper surface seed disinfection. 

To isolate endophytic bacteria, surface-disinfected seeds were macerated in a sterile mortar with 1 mL of a saline solution (0.9% NaCl). One hundred microliters of the suspension were cultured in three different media: TY, R2A (0.5 g·L^−1^ yeast extract, 0.5 g·L^−1^ protease peptone, 0.5 g·L^−1^ casein acidic peptone, 0.5 g·L^−1^ glucose, 0.5 g·L^−1^ soluble starch, 0.3 g·L^−1^ K_2_HPO_4_, 0.05 g·L^−1^ MgSO_4_*7H_2_O, 0.3 g·L^−1^ sodic pyruvate, 15 g·L^−1^ agar; pH 7.2) and a yeast–mannitol agar (10.0 g·L^−1^ mannitol, 0.5 g·L^−1^ yeast extract, 0.5 g·L^−1^ K_2_HPO_4_, 0.2 g·L^−1^ MgSO_4_*7H_2_O, 0.10 g·L^−1^ NaCl, 0.15 g·L^−1^ CaCl_3_, 15 g·L^−1^ agar; pH 6.8). Cultures were incubated for three days at 30 °C, and then colonies with different colors and morphologies were selected and purified. Isolates were stored at −20 °C with 20% (*v*/*v*) glycerol for further studies.

The bacterial isolates were identified by the sequence analysis of almost the entire *16S rRNA* gene, as described by Mareque et al. [28]. The universal primers Eub27f (5-AGAGTTTGATCMTGGCTCAG-3) and Eub1492r (5-TACGGYTACCTTGTTACGACTT-3) were used for PCR amplification. The quality of sequences was checked in the FinchTV program (v.1.4.0) (Geospiza, Inc., Denver, CO, USA) using a quality value equal to or greater than 20 per base as an acceptance criterion. Sequences were assembled using the DNA Baser Sequence Assembler software v. 4.10.1.13 (2012, Heracle BioSoft SRL, http://www.dnabaser.com; accessed on 26 April 2023). For isolate identification at the genera level, the closest type of strain was determined, and a phylogeny was constructed. The closest type strains were obtained by comparing the consensus sequences with the Ribosomal Database Project (RDP) (http://rdp.cme.msu.edu/seqmatch/seqmatch_intro.jsp; accessed on 26 April 2023). For phylogenetic analysis, the sequence alignments were performed using the ClustalW multiple sequence alignment tool in MEGA-X software (v. 10.0.4) [29,30]. Phylogenetic trees were constructed with the neighbor-joining method using the default parameters in the MEGA-X software.

### 2.2. Screening of Plant Growth-Promoting Traits

#### 2.2.1. Indolic Compound Production

Production of indolic compounds was assessed in triplicate using the Salkowski method (λ = 540 nm) [31] in a VarioskanFlash v. 4.00.51 spectrophotometer (Thermo Scientific, Waltham, MA, USA). Tryptophan (200 μg·mL^−1^) was used as an inductor to synthesize indolic compounds. Production was detected as a pink coloration of the medium after 72 h of incubation at 30 °C. *Gluconoacetobacter diazotrophicus* strain Pal5 was used as a positive control [32]. A positive value was considered when the three replicates of the tested isolates had a calculated threshold value greater than 10 µg·mL^−1^ of indolic compounds.

#### 2.2.2. Phosphate and Potassium Solubilizing Capabilities

The phosphate solubilization capability of the isolates was evaluated in vitro. Ten microliters of a suspension of the optical density of 0.2 at 620 nm were spotted in triplicate in glucose a GL-rich medium supplemented with calcium phosphate (10.0 g·L^−1^ glucose, 2.0 g·L^−1^ yeast extract, 50 mL·L^−1^ of 10% K_2_HPO_4_, 100 mL·L^−1^ of 10% CaCl, 15.0 g·L^−1^ agar; pH 5.8) and on National Botanical Research Institute’s Phosphate (NBRIP) medium (10.0 g·L^−1^ glucose, 5.0 g·L^−1^ Ca_3_(PO_4_)_2_, 5.0 g·L^−1^ MgCl_2_*6H_2_O, 0.25 g·L^−1^ MgSO_4_*7H_2_O, 0.2 g·L^−1^ KCl, 0.1 g·L^−1^ (NH_4_)_2_SO_4_, 18.0 g·L^−1^ agar; pH 6.8) [25,33]. Aleksandrov medium (10.0 g·L^−1^ sucrose, 1.5 g·L^−1^ K_2_HPO_4_, 0.5 g·L^−1^ MgSO_4_*7H_2_O, 1.0 g·L^−1^ CaCO_3_, 15.0 g·L^−1^ agar; pH 7.5) was used to evaluate potassium phosphate solubilization [34]. A translucent halo around the colony after 72 h in three replicates was interpreted as positive to solubilization. Translucent halos on the NBRIP medium were measured (cm), and the solubilizing index was calculated using the following formula: SI = TD/CD; where SI is the solubilization index, DT is the total diameter (cm), and CD is the colony diameter (cm) [35]. *Pantoea* sp. strain UYSB45 was used as a positive control [28].

#### 2.2.3. Siderophore Production

The chromeazurol (CAS) agar assay was used to detect siderophore production [36]. Aliquots of 10 μL of bacteria inoculum were put on the medium. In this assay, a yellow halo around the colony indicates siderophore production. *Herbaspirillum seropedicae* strain Z67 was used as a positive control [37]. 

#### 2.2.4. Production of Hydrolytic Enzymes

Hemicellulose, cellulose, and protease hydrolytic activities were evaluated with three replicates in plates containing a TY or TSA solid medium supplemented with 0.5% (*w*/*v*) Avicel, 0.2% (*w*/*v*) carboxymethyl cellulose, or 10% (*w*/*v*) of skimmed milk, respectively [25]. Enzymatic activities were detected qualitatively by the presence of a halo around the colony after 48 h of incubation at 30 °C. *Acinetobacter* sp. strain UYSB41 was used as a positive control in hemicellulose and cellulose hydrolysis assays [28]. *Pseudomonas* sp. strain UYFA214 was used as a positive control in protease activity assays [38].

#### 2.2.5. Biofilm Formation

Biofilm formation was detected using the crystal violet (CV) staining method. The isolates were grown with agitation in 96-well plates until an optical density of 0.2 at 620 nm. The plates were incubated for 48 h at 30 °C without agitation, and the supernatant was removed. Then, the plates were washed with phosphate-buffered saline, and the CV solution (0.1% *w*/*v*) was added and then incubated for 20 min. The CV excess was removed with water, while the bound CV was released from the cells by adding ethanol. The suspension absorbance was measured at 570 nm using a VarioskanFlash v. 4.00.51 spectrophotometer (Thermo Scientific). 

### 2.3. Biocontrol Activity against Pyricularia oryzae

According to the screening of plant growth-promoting traits, the more promising bacterial strains were used to assess further antagonistic abilities against the fungal phytopathogen *P. oryzae* strain 28 (Micoteca, UCTB-LP, INCA). Antagonism was evaluated in dual culture on a PDA medium (National Bioprepareds Center Bejucal, Cuba), as previously reported [25]. Plates with the PDA medium inoculated only with the fungus were used as a negative control. The percentage of radial growth inhibition of *P. oryzae* was evaluated after 13 and 16 days of incubation through the formula: PRGI = (1 − r1/r2) × 100, where PRGI is the percentage of radial growth inhibition of *P. oryzae*, r1 is the radial growth of *P. oryzae* in Petri dishes with bacteria strains, and r2 is the radial growth of *P. oryzae* in Petri dishes without bacteria strains [39]. 

### 2.4. Effect of Bacterial Inoculation in Rice Growth in Hydroponic System

Seeds of *O. sativa* cultivars INCA LP-5 and INCA LP-7 were surface-disinfected [27]. Surface-disinfected seeds were placed in Petri dishes containing 0.8% (*w*/*v*) agar in water and incubated in the dark at 30 °C for three days. Fifteen seedlings were sown into a (800 mL) pot (diameter 8.3 cm, height 14.5 cm) containing 50 mL of diluted Hoagland nutrient solution (1:2). Bacteria were grown in TY media at a concentration of 5 × 10^10^ CFU mL^−1^. Then, 0.5 mL of the inoculant was mixed with diluted Hoagland nutrient solution for a final concentration of 5 × 10^8^ CFU mL^−1^ in each pot. A negative control without bacterial inoculation was included. *Herbaspirillum seropedicae* strain Z67 was used as a positive control [37]. 

The pots were covered with translucent nylon and were located in controlled conditions following a randomized design and using 30 seedlings per treatment (two pots with 15 seedlings per treatment). The seedlings were grown with a photoperiod of 12 h light/12 h darkness conditions at 26 °C/22 °C (day/night) and 70% relative humidity for 21 days, and at this moment, the seedlings were harvested. The height and root length (cm), shoot and root fresh weight (mg), and shoot and root dry weight (mg) (dried at 70 °C until constant weight) were determined.

### 2.5. Statistical Analysis

Data from the screening for phosphate solubilizing abilities, percentage of radial growth inhibition of *P. oryzae*, and the rice growth in the hydroponic system were subjected to normality (Bartlett test) and variance homogeneity (Kolmogorov–Smirnov test). Simple classification analysis of variance (ANOVA) was applied, with the Tukey HSD mean comparison test for *p* < 0.05. Statgraphic Plus v. 5.0 software was used for statistical data processing, and Microsoft Excel 2010 software for its representation.

## 3. Results

### 3.1. Identification of Seed Endophytic Bacteria from Rice Cv. INCA LP-5 and INCA LP-7

**Figure 1:**
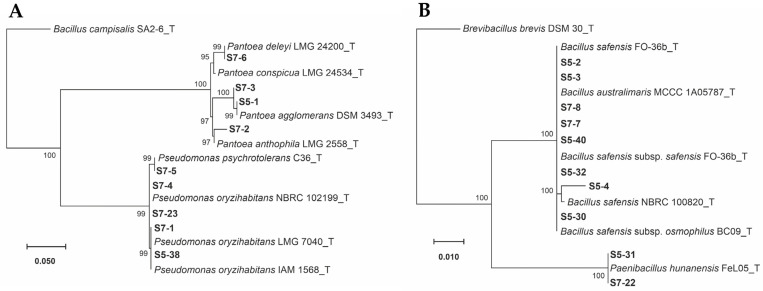
Maximum likelihood phylogenetic trees based on *16S rRNA* partial sequences of bacteria isolated from rice seed (*Oryza sativa* L.) cultivars INCA LP-5 and INCA LP-7. The Tamura–Nei model with a discrete Gamma distribution was used. Numbers at branches bootstrap values of >70% from 1000 replicates. Scale bar number of nucleotide substitutions per site. (**A**) Phylogenetic tree corresponding to the Gram-negative isolates. *Bacillus campisalis* strain SA2-6 was used as the outgroup; (**B**) phylogenetic tree corresponding to the Gram-positive isolates. *Brevibacillus brevis* strain DSM 30 was used as the outgroup.

Nineteen isolates were obtained from surfaced disinfected seeds of rice cv. INCA LP-5 and INCA LP-7. All the isolates were obtained from seeds with the husk, while none were from disinfected seeds without the husk. Nine of them were isolated from the seeds of rice cv. INCA LP-5 and ten from cv. INCA LP-7. The strains’ identification showed that 22.2% (two strains) and 70% (seven strains) of the isolates from INCA LP-5 and INCA LP-7 seeds, respectively, were Gram-negative and belonged to the phylum Pseudomonadota. They were assigned to the genera *Pantoea* and *Pseudomonas* (Figure 1A, Table 1). The 77.8% (seven strains) and 30% (three strains) of the remainder strains, respectively, were Gram-positive and belonged to the phylum Bacillota. They were related to the genera *Bacillus* and *Paenibacillus* (Figure 1B, Table 1).

The phylogenetic analyses showed that three strains belonged to the genus *Pantoea* and formed a well-supported cluster related to some types of strains of *Pantoea* (Figure 1A). In the first group, strains S7-6 had a 99.85% sequence identity with *Pantoea deleyi* LMG 24200. In contrast, strains S7-3 and S5-1 belonged to the second group and had a 97.70% and 97.50% sequence identity with *Pantoea agglomerans* DSM 3493. Strain S7-2 from the third group was closely related to *Pantoea anthophila* LMG 2558 (Table 1, Figure 1A). Within the strains belonging to the *Pseudomonas* genera, the results showed strain S7-5 was closely related to *Pseudomonas psychrotolerans* C36 (Figure 1A). The other four strains, S7-4, S7-23, S7-1, and S5-38, were related to the *Pseudomonas* genus and were closely related to *Pseudomonas oryzihabitans* (Figure 1A) (Table 1). Similarly, strains S5-2, S5-3, S7-8, S7-7, S5-40, S5-32, S5-4, and S5-30 belonged to the genus *Bacillus* and were closely related to *Bacillus safensis* and *Bacillus australimaris* (Figure 1B; Table 1). Only strains S5-31 and S7-22 belonged to the genus *Paenibacillus* and formed a well-supported cluster related to *Paenibacillus* (Figure 1B) with a 99.72% and 97.79% sequence identity with *Paenibacillus hunanensis* FeL05, respectively (Table 1). Interestingly, strains S7-3 (from INCA LP-7) and S5-1 (from INCA LP-5) formed a well-supported cluster with *P*. *agglomerans* DSM 3493, strains S7-1 and S5-38 with *P. oryzihabitans* LMG 7040, and strains S7-22 and S5-31 with *P. hunanensis* FeL05 (Table 1).

### 3.2. Screening of Plant Growth-Promoting Traits 

Several bacterial strains isolated from the rice seeds cv. INCA LP-5 and INCA LP-7 have phytostimulation, biofertilization, plant infection, and biocontrol capabilities (Table 2). The results showed that 57.9% of the bacterial strains produced indolic compounds in the culture media with tryptophan. Interestingly, most of the strains presented at least one plant growth-promoting trait. In that sense, 42.1% and 21.1% of the strains solubilized calcium phosphate in the NBRIP and GL media, respectively. The halo produced by *Pseudomonas* sp. strain S5-38 was higher in the NBRIP medium (Table 2). Solubilization of potassium was observed in 21.1% of the strains. Furthermore, 36.8% of the strains were able to produce siderophores (Table 2). Concerning abilities putatively involved in plant infection, 21.1%, 15.8%, and 52.6% of strains presented exo-cellulase, hemicellulase, and protease activities, respectively. Furthermore, 26.3% of the strains formed biofilms (Table 2).

The isolate *Pseudomonas* sp. strain S5-38 and *Pantoea* sp. strain S7-3 were further selected for an antagonism assay against *P. oryzae* since they practically presented all the traits putatively involved in phytoestimulation and biofertilization, and some are involved in infection attributes. Furthermore, the strains belonged to different genera. The results showed that both strains inhibited fungal growth. However, the strain *Pantoea* sp. strain S7-3 produced a higher inhibition of fungi growth than the *Pseudomonas* sp. strain S5-38 after 13 and 16 days (14.7–54.1%; Table 2).

### 3.3. Selected Seed Endophytes Promote the Rice Growth

**Figure 2:**
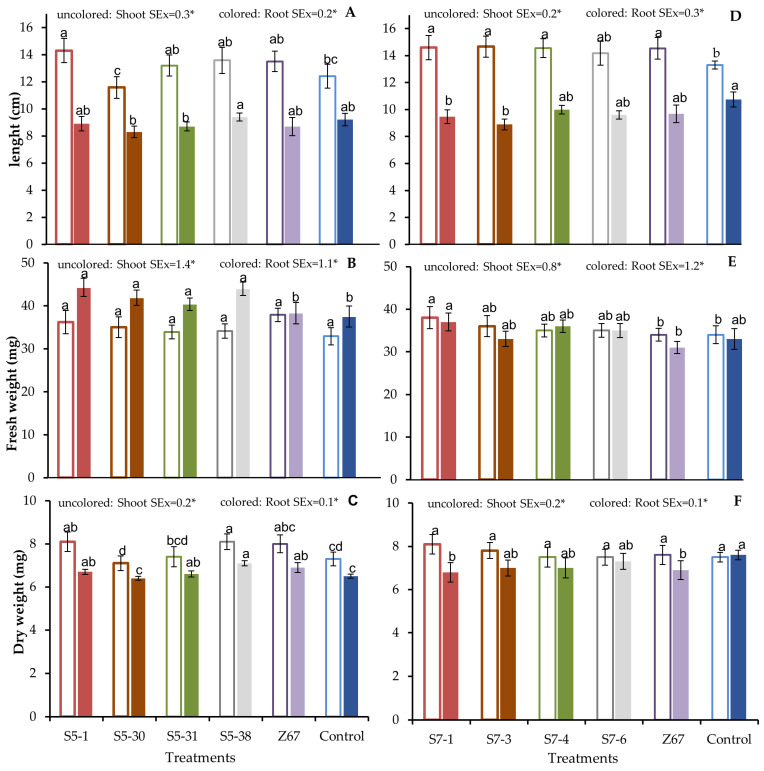
Effects of inoculation with putative seed bacterial endophytes previously selected and *Herbaspirillum seropedicae* strain Z67 (reference strain) on the growth of rice cv. INCA LP-5 (**A**–**C**) and cv. INCA LP-7 (**D**–**F**), 21 days post-inoculation (dpi) in in vitro conditions. The control treatment was the plants inoculated with the sterile TY medium. The data reported are the means of thirty sample replicates. Different letters mean that the difference between treatments is significant (Tukey HSD *p* < 0.05, *n* = 30). Uncolored bar represents the plant shoot; colored bar, plant root. SEx, standard errors of ANOVA; (*) significance values.

The results obtained from the plant assays showed that *O. sativa* cv. INCA LP-5 plants inoculated with the *Pantoea* sp. strain S5-1, which increased their height compared to the non-inoculated plants (Figure 2A). Furthermore, the treatments inoculated with the *Pantoea* sp. strain S5-1 and *Pseudomonas* sp. strain S5-38 increased the shoot and root dry weight of the rice plants and were more significant than those inoculated with the *H. seropedicae* strain Z67 (the reference strain) in the root dry weight (Figure 2B,C). The treatments did not affect the root length and shoot fresh weight of *O. sativa* cv. INCA LP-5 plants.

In the assay with *O. sativa* cv. INCA LP-7, the plants inoculated with the *Pseudomonas* sp. strain S7-1, *Pantoea* sp. strain S7-3, *Pseudomonas* sp. strain S7-4, and reference strain increased the plant height in comparison to the non-inoculated plants (Figure 2D). Furthermore, the shoot fresh weight was increased only with the inoculation of the *Pseudomonas* sp. strain S7-1. The plants inoculated with this strain were greater than those inoculated with the reference strain in the shoot and root fresh weights (Figure 2E). The inoculation did not affect the shoot and root dry weights (Figure 2F; Dataset S1).

## 4. Discussion

In this study, we isolated culturable bacterial endophytes from the Cuban rice cultivars INCA LP-5 and INCA LP-7 seeds and explored their potentiality as PGPB. Interestingly, we found that all strains came from disinfected seeds with the husk, while we could not detect bacterial growth from the macerates of seeds without the husk. Therefore, our results indicate that the strains were localized between the husk and the endosperm. Nonetheless, in a similar study, when the husks of rice seeds were removed, the presence of different culturable bacteria was found in the seed’s endosphere [21]. We cannot discard the possibility of other bacteria that could not grow in the culture media used in our study. In that sense, it has been reported that many endophytic bacteria are now being identified and characterized by culture-independent methods, and only a few of them are culturable, including some rice seed endophytes [21,40]. Culture-independent methods such as metabarcoding analysis could reveal the presence of other bacteria associated with the rice cultivars INCA LP-5 and INCA LP-7 seeds.

The bacterial community associated with the rhizosphere of cultivars INCA LP-5 and INCA LP-7 has been previously studied [25,26]. However, this is the first study describing the presence of bacteria in the seeds of both cultivars. Bacterial seed endophytes are essential in maintaining plant health in the early stages, participating in nutrient mobilization, and promoting plant growth [41,42]. Thus, isolating and characterizing bacterial endophytes from seeds could contribute to selecting promising strains to promote rice growth. A previous study analyzed the bacterial population profiles in the seeds of salt-tolerant and salt-sensitive cultivars of *O. sativa* ssp. Gram-negative bacteria were more abundant in salt-tolerant cultivars, while Gram-positive bacteria were more abundant in salt-sensitive cultivars [21]. Our results agree with this observation since INCA LP-7 (salt-tolerant) seeds have a higher percentage of Gram-negative culturable strains. In contrast, Gram-positive strains were prevalent in INCA LP-5 (salt-sensitive) seeds.

It is conceivable that plants maintain a core microbiome independent of the soil type, environment, host genotype, and agricultural management [43]. In our study, the genera *Bacillus, Pantoea*, *Pseudomonas,* and *Paenibacillus* were identified as part of the bacterial cultivable community found in the rice seeds of both cultivars (Table 1, Figure 1). All of these genera had been reported as the seeds’ rice endophytes, which suggests the existence of a core microbiome consistently associated with rice plants regardless of location, genotype, and harvesting time [21,44,45,46]. This fact suggests the possibility of developing and applying universal microbial inoculants to benefit global rice production. A previous study showed that around 64% of culturable strains from the rhizosphere of the rice cultivar INCA LP-7 belonged to the genus *Pantoea* [26]. Our result showed the presence of *Pantoea* as the bacterial endophytes of rice seeds from the cultivar INCA LP-7. The rhizosphere is a source of plant bacterial endophytes, and some of them can colonize the seeds. Another pathway to achieve the seed is via the stigma of the mother plant, explaining the presence of these bacterial communities inside the rice seeds [47]. Some of the obtained bacterial endophytes isolated from the rice seed cultivars INCA LP-5 and INCA LP-7 have potentialities as PGPB since they harbor traits related to direct growth-promoting mechanisms such as indole acetic acid (IAA) production and phosphate and potassium solubilization, as well as siderophore production (Table 2). Other experiments conducted on rice seed endophytes, including strains belonging to *Bacillus* and *Pseudomonas* genera, have also shown the presence of these attributes [21,45,48]. Endophytic microbes that produce IAA have a significant role in mutualistic interactions between the host plant and endophytes and, hence, regulate plant growth [49,50]. Some of the isolated strains produce this kind of compound in vitro in the presence of tryptophan. This amino acid is a physiological precursor to auxin production in microorganisms and plants. Previously, the efficacy of endophytic bacteria in producing high IAA concentrations was demonstrated and significantly improved with increasing tryptophan levels. Endophytic bacteria isolated from the leaves of *Pulicaria incisa* can regulate plant growth through IAA synthesis [51].

The capability of the *Pseudomonas* sp. strain S5-38, *Pantoea* sp. strain S7-3, *Pantoea* sp. strain S7-6, and *Pseudomonas* sp. strain S7-23 to solubilize potassium can help to minimize chemical fertilizer application in rice plants. Using beneficial bacteria as bio-inoculants increased the grain yield and the efficient use and uptake of potassium in straw and grains [52]. Despite potassium being one of the most essential macronutrients, its uptake is often limited due to its low bioavailability in natural conditions [53]. Therefore, seed endophytic strains that can improve potassium availability are critical for improving rice growth under challenging environments. In the same line, siderophores are secondary metabolites that can be synthesized by endophytic bacteria and have the ability to iron-chelate from complex substances [54]. In the current study, bacteria belonging to *Pseudomonas* and *Pantoea* genera can synthesize siderophores (Table 2). These results agree with recent studies analyzing the capability of bacterial endophytes isolated from the roots of *Thymus vulgaris* belonging to the *Bacillus* genera to produce these compounds [55].

The capability of some of the isolated strains to form biofilms can be critical to sustaining beneficial functions that benefit plant growth, including the production of phytohormones and nutrient solubilization (Table 2). Previous studies have demonstrated some endophytic *Burkholderia* and *Azotobacter* strains form biofilms during the colonization of rice plants [56,57]. Furthermore, it was known that biofilm formation allows bacteria to be more resistant to environmental stresses or microbial deleterious substances. The rice endophyte *G. diazotrophicus* produces exopolysaccharide, essential for biofilm formation, which is a critical process for colonizing essential plant organs [58]. 

The biocontrol of fungal phytopathogens is an indirect mechanism by which symbiotic bacteria can promote plant growth. In this study, we found that *Pseudomonas* and *Pantoea* inhibited the growth of the phytopathogenic fungus *P. oryzae* (Table 2). Bacterial seed endophytes from the genera *Pseudomonas, Pantoea, Paenibacillus,* and *Bacillus* isolated from rice seeds have shown antifungal activity against some phytopathogenic fungi such as *Curvularia* sp., *Fusarium oxysporum*, *Pythium ultimum*, *Rhizoctonia solani*, and *Pyricularia grisea* [59,60,61]. The secretion of hydrolytic enzymes and siderophores production are mechanisms that use PGPB to inhibit the filament fungus growth as they break down the glycosidic bonds of chitin of the cell wall of the fungus and to establish competence for iron, respectively [62]. This agrees with our results in which endophytic bacteria such as the *Pseudomonas* sp. strain S5-38 and *Pantoea* sp. strain S7-3 demonstrated both inhibition of fungal growth and the production of hydrolytic enzymes (Table 2). 

To know the effect of the more promising bacteria on plant growth, eight strains were selected and inoculated on Cuban rice cultivars. The application of seed endophytic bacteria for promoting plant growth is gaining importance due to endophyte beneficial effects and their environment. Despite some of the isolated bacterial genera that have been previously reported on promoting the seedling development of rice, this is the first time reporting seed endophytes from Cuban rice cultivars that promote plant growth. In this study, we highlight *Pantoea* sp. S5-1, as this strain enhances practically all the variables evaluated (the plant height, root fresh weight, shoot dry weight, and root dry weight) in the rice seedlings cv. INCA LP-5 (Figure 2). The promoter activity of this genus in rice plants is known since it solubilizes inorganic phosphate and produces indol acetic acid and siderophores [21,63,64]. Furthermore, some strains belonging to the genera *Pseudomonas* promote rice growth (Figure 2). Recently, *Pseudomonas* isolated from maize rhizospheres altered the root architecture and enhanced rice growth and yield [65,66]. Despite this study identifying only the culturable bacterial diversity inhabiting the seeds of Cuban rice, obtaining culturable strains and exploring their plant growth-promoting capabilities is critical for designing plant biostimulants. Therefore, although this study did not allow us to know the total bacterial communities in rice seeds, it is an ecological approach for identifying the plant growth-promoting traits of seed endophytes interacting with Cuban rice during the early stages of plant development. 

## 5. Conclusions

This study isolated and characterized endophytic bacteria associated with rice seeds, providing valuable insights into the bacterial communities linked to two of Cuba’s most significant rice cultivars. These results mark a pioneering investigation in the country, revealing the potential of nineteen strains as biofertilizers, biostimulants, and biocontrol agents, some of which effectively promote rice growth. This is of particular importance, considering that seed endophytes serve as the initial inoculum for the plant. Notably, strains like *Pantoea* sp. S5-1, *Pseudomonas* sp. S5-38, and *Pseudomonas* sp. S7-1 can be selected for the development of biostimulants aimed at enhancing rice crop growth. 

## Figures and Tables

**Table 1:** *16S rRNA* nucleotide sequence similarities of putative endophytic bacterial isolates from surface-disinfected seeds of rice plant (*O. sativa* L.) cv. INCA LP-5 and INCA LP-7.

Rice Cultivar	Isolate	Accession Number	Best Hit (Accession Number) ^a^	Maximum Identity (%)
INCA LP-5	S5-1	MT808964	*Pantoea agglomerans* (KY013009.1)	97.50
	S5-2	MT808965	*Bacillus safensis* (NR_041794.1)	99.93
	S5-3	MT808966	*Bacillus australimaris* (MN077148.1)	99.86
	S5-4	MT808967	*B. safensis* (NR_041794.1)	100
	S5-30	MT808968	*B. australimaris* (MN077148.1)	99.86
	S5-31	MT808969	*Paenibacillus hunanensis* (NR_116440.1)	99.72
	S5-32	MT808970	*B. australimaris* (MN077148.1)	100
	S5-38	MT808971	*Pseudomonas oryzihabitans* (NR_115005.1)	99.85
	S5-40	MT808972	*B. australimaris* (MN077148.1)	99.86
INCA LP-7	S7-1	MT808973	*P. oryzihabitans* (NR_114041.1)	98.78
	S7-2	MT808974	*Pantoea ananatis* (LC462185.1)	98.36
	S7-3	MT808975	*P. agglomerans* (KY013009.1)	97.70
	S7-4	MT808976	*P. oryzihabitans* (NR_114041.1)	99.93
	S7-5	MT808977	*P. oryzihabitans* (NR_115005.1)	100
	S7-6	MT808978	*Pantoea deleyi* (NR_116114.1)	99.85
	S7-7	MT808979	*B. australimaris* (MN077148.1)	99.86
	S7-8	MT808980	*B. australimaris* (MN077148.1)	99.77
	S7-22	MT808981	*P. hunanensis* (NR_116440.1)	97.79
	S7-23	MT808982	*Pseudomonas psychrotolerans* (NR_042191.1)	100

^a^ According to the Ribosomal Database Project (RDP).

**Table 2:** Plant growth promotion features of selected putative endophytic bacterial isolates from superficially disinfected seeds of rice plants (*Oryza sativa* L.) cv. INCA LP-5 and INCA LP-7. Values are the mean of three replicates (*n* = 3). Same letter follow means the difference is not significant in the same column (Tukey HSD *p* < 0.05).

Isolate	Phytostimulation and Biofertilization ^a^					Infection ^b^				Antagonism against *P. oryzae* ^c^	
	Ind. Com.	Ca_3_PO_4_ Solubilization		K	SID	Lithic Enzymes			Biofilm	13 dpi	16 dpi
						*EC*	*HC*	*PROT*			
		NBRIP	GL								
S5-1	+	1.3 cd	−	−	+	−	−	−	+	ND	ND
S5-2	*+*	−	−	−	−	−	−	+	−	ND	ND
S5-3	−	−	−	−	−	−	−	+	−	ND	ND
S5-4	−	−	−	−	−	−	−	+	+	ND	ND
S5-30	*+*	1.7 b	−	−	−	−	−	+	−	ND	ND
S5-31	+	1.6 bc	−	−	−	+	−	+	−	ND	ND
S5-32	−	−	−	−	−	−	−	+	−	ND	ND
S5-38	+	2.1 a	+	+	+	+	−	−	+	14.7 b	27.5 b
S5-40	−	−	−	−	−	−	−	+	−	ND	ND
S7-1	+	1.5 bc	−	−	+	−	−	−	+	ND	ND
S7-2	+	−	−	−	+	−	+	−	−	ND	ND
S7-3	+	1.3 cd	−	+	+	+	−	−	−	46.1 a	54.1 a
S7-4	+	1.1 d	+	−	+	−	−	−	−	ND	ND
S7-5	+	−	+	−	−	−	−	−	−	ND	ND
S7-6	+	1.4 bcd	−	+	−	−	+	−	−	ND	ND
S7-7	−	−	−	−	−	−	−	+	+	ND	ND
S7-8	−	−	−	−	−	−	−	+	−	ND	ND
S7-22	−	−	−	−	−	+	+	+	−	ND	ND
S7-23	−	−	+	+	+	−	−	−	−	ND	ND

^a^ Ind. Comp, indolic compounds; phosphate solubilization in GL and NBRIP medium (cm); K, potassium solubilization in Alexsandrov medium; SID, siderophore detection in CAS solid medium; ^b^ EC, exo-cellulase activity; HC, hemicellulose activity; PROT, protease activity; ^c^ 13 days pi, 16 days pi, percentage of *Pyricularia oryzae* growth inhibition at 13 and 16 days post inoculation, respectively. ND = not detected.

## Data Availability

The 16S rRNA sequences from the obtained isolates were submitted to the GenBank database under accession numbers MT808964 to MT808982.

## Supplementary Materials

The following supporting information can be downloaded at: https://www.mdpi.com/article/10.3390/microorganisms11092317/s1. Dataset S1: Effect of bacterial inoculation on rice growth in a hydroponic system.
